# Echinococcosis, Ningxia, China

**DOI:** 10.3201/eid1108.041179

**Published:** 2005-08

**Authors:** Yu Rong Yang, Tao Sun, Zhengzhi Li, Xiuping Li, Rui Zhao, Li Cheng, Xiao Pan, Philip S. Craig, Dominique A. Vuitton, Donald P. McManus

**Affiliations:** * Queensland Institute of Medical Research, Brisbane, Queensland, Australia;; † Ningxia Medical College, Ningxia Hui Autonomous Region, China;; ‡ The Second Provincial Hospital of Ningxia Hui Autonomous Region, Ningxia, China;; § Xiji County Hospital, Xiji, Ningxia Hui Autonomous Region, China;; ¶ University of Salford, Salford, United Kingdom;; # WHO Collaborating Centre for Prevention and Treatment of Human Echinococcosis, Besancon, France

**Keywords:** Ningxia Hui Autonomous Region, alveolar echinococcosis, cystic echinococcosis

**To the Editor:** Ningxia is the smallest provincial autonomous region on the Loess Plateau in central China, with a population of ≈5.5 million persons composed of 35 ethnic and various religious groups. The natural environmental conditions and socioreligious ethnic status in Ningxia, particularly the southern region, are conducive to sheep farming, an important part of the agricultural economy. Both cystic echinococcosis (CE) and alveolar echinococcosis (AE) (*1*) are endemic in the northwest part of China; high prevalences have been reported in several provinces (*1*), including Gansu (*2**,**3*) and the Xinjiang Uigur Autonomous Region (*4*). Little information is available about the extent of human echinococcosis in Ningxia; this is the first report of a provincial investigation for both human CE and AE there.

We conducted a retrospective survey of clinical records from 7 local county hospitals and 4 other hospitals in Yinchuan to determine the epidemiology of human echinococcosis in southern Ningxia. All surgical and clinical records were checked, and diagnoses were confirmed based on imaging, surgical reports, and histopathologic reports. Data concerning age, sex, domicile, ethnicity, occupation, year of diagnosis, cyst or lesion numbers and anatomical location, type and duration of anthelminthic treatment (if given), and the nature and number of surgeries performed for echinococcosis were recorded for each confirmed CE and AE patient.

From 1985 to 2002, a total of 2,216 cases of echinococcosis were recorded, most of which were due to CE (96%). The incidence of combined CE and AE from 1994 to 2001 was 7/100,000 persons for southern Ningxia, compared with 1/100,000 persons for Yinchuan in the north (Table). Human AE cases were reported only from the 3 southern counties of Xiji, Haiyuan, and Guyuan; CE cases occurred throughout Ningxia, with a mixed endemic focus for both human CE and AE in the south. A variable distribution of AE and CE cases from 1994 to 2001 was evident from hospital records. Both AE and CE were recorded in Xiji (6/100,000 persons), Guyuan (5/100,000 persons), and Haiyuan (11/100,000 persons) counties. CE incidences for Tongxin (13/100,000 persons) and Pengyang (5/100,000 persons) were substantially higher than for Longde (0.5/100,000 persons) and Jingyuan (0.15/100,000 persons). This heterogeneous distribution of echinococcosis may reflect different patterns of parasite transmission in different areas of Ningxia. CE and AE incidence (Table) were compared for 1994 and 2001 by chi-square test. Apart from a substantial increase in Haiyuan (p<0.05) and a substantial decrease in Xiji (p<0.05), no other substantial incidence changes were apparent (Table).

**Table:** Average incidence of human echinococcosis (AE/CE combined), 1994–2001, and incidence compared for ethnicity (Han/Hui) in southern Ningxia and Yinchuan City*

Location (population)	1994–2001		Ethnicity comparison							
			Hui (1994–2001)			Han (1994–2001)				
	Cases	Incidence†	%‡	Cases	Incidence†	%‡	Cases	Incidence†	IRR	95% CI
Ningxia										
Tongxin (328,700)	353	13.42	81.49	291	13.58	18.48	62	12.75	0.94	0.71–1.25§
Haiyuan (344,000)	305	11.08	70.58	195	10.04	28.52	110	14.01	1.40	1.10–1.77
Guyuan (470,700)	226	6.00	43.23	108	6.63	56.70	118	5.52	0.83	0.64–1.09§
Xiji (403,100)	198	6.14	52.00	89	5.30	47.99	109	7.04	1.33	0.99–1.77§
Pengyang (231,000)	101	5.46	29.77	46	8.36	70.23	55	4.24	0.51	0.34–0.76
Longde (197,300)	8	0.50	8.98	1	0.70	91.01	7	0.53	0.76	0.1–16§
Jingyuan (81.301)	1	0.15	96.50	1	0.15	3.48	0	0	—	§
Subtotal (2,056,101)	1,192	7.24	—	—	—	—	—	—	—	—
Yinchuan (1,614,700)	165	1.27	18.36	59	2.48	79.88	106	1.02	0.41	0.3–0.57
Total (3,670,801)	1,357	4.62	33.88	790	7.94	65.47	567	2.94	0.37	0.33–0.41

*AE, alveolar echinococcosis; CE, cystic echinococcosis; IRR, incidence risk ratio; CI, confidence interval. †Cases/100,000/year. ‡Ethnicity composition ratio (%) at each location. §Comparison between ethnicity composition ratio and incidence not significant.

Patients' ages ranged from 1 to 80 years for men and from 3 to 77 years for women (mean age 35.7 years). The patient sex ratio was 0.72 (916 men, 1,268 women), whereas the population sex ratio was 1.05, indicating significant differences in echinococcosis case numbers between men and women (p<0.05). Farm laborers accounted for 66.1% (1,464/2,216) of cases, students 12.4% (275/2,216), workers and self-employed 5.2% (116/2,216), village leaders 4.8% (106/2,216), teachers and housewives 0.9% (21/2,216), butchers 0.2% (4/2,216), and others 0.1%. A comparison of the ethnic composition ratio (*5*) with an average incidence from 1994 to 2001 showed a substantial difference between the Hui and Han nationalities in Yinchuan, Haiyuan, and Pengyang, and for Ningxia as a whole. No substantial difference was shown in Longde, Tongxin, Guyuan, and Xiji for the 2 ethnic groups by incidence risk ratios with 95% confidence intervals (Table).

Radical surgery to remove CE cysts was performed for 83.3% of patients; only 7.3% of patients received combined albendazole or mebendazole chemotherapy pre- and post-surgery. Recurrences of CE cysts that required surgery were high (30%), suggesting inadequate surgical and medical care. Most AE patients received only chemotherapy treatment and were at an advanced stage of infection.

The retrospective data of human AE and CE infection rates in the current study suggest that disease transmission was more pronounced 10–15 years earlier. The landscape of woods and scrub cover was greater then, domestic sheep and dog populations were larger, and red fox and rodent species densities were likely higher, providing optimal transmission conditions for both *Echinococcus granulosus* and *E. multilocularis*.

The incidence data (2001) for human CE and AE are likely underestimated for several reasons. Access to medical treatment for villagers is problematic and many asymptomatic cases (*6*) would be excluded. An age and sex bias also exists, as generally older persons and women receive most of the limited medical attention. Furthermore, the high surgical costs for treatment (CE and AE), especially in the past 10 years, coupled with poor economic development, preclude access to treatment in many rural communities. Because abdominal ultrasound examinations for CE and AE can identify clinically silent infections (*7**,**8*), comprehensive community-based studies are needed to identify asymptomatic or early-stage cases.

Our study provides accurate data to determine the true prevalence and incidence of CE and AE, which contributes to better treatment outcomes. These data can help determine the reasons for the heterogeneity of disease distribution in Ningxia to better prepare for future echinococcosis control strategies throughout the region.

## References

[1] Vuitton DA, Zhou H, Bresson-Hadni S, Wang Q, Piarroux M, Raoul F, Epidemiology of alveolar echinococcosis with particular reference to China and Europe. Parasitology. 2003;127(Suppl):S87–107. 10.1017/S0031182003004153 15027607

[2] Craig PS, Giraudoux P, Shi D, Bartholomot B, Barnish G, Delattre P, An epidemiological and ecological study of human alveolar echinococcosis transmission in south Gansu, China. Acta Trop. 2000;77:167–77. 10.1016/S0001-706X(00)00134-0 11080507

[3] Bai Y, Cheng N, Jiang C, Wang Q, Cao D. Survey on cystic echinococcosis in Tibetans, West China. Acta Trop. 2002;82:381–5. 10.1016/S0001-706X(02)00038-4 12039679

[4] Wang YH, Rogan MT, Vuitton DA, Wen H, Bartholomot B, Macpherson CN, Cystic echinococcosis in semi-nomadic pastoral communities in north-west China. Trans R Soc Trop Med Hyg. 2001;95:153–8. 10.1016/S0035-9203(01)90142-7 11355546

[5] Qu Y, Sun ZW. Atlas of Ningxia Hui Autonomous Region. Ying S, editor. Yinchuan: Publishing House of Xi'an Atlas; 2000. p. 90–130.

[6] Craig PS, Rogan MT, Allan JC. Detection, screening and community epidemiology of taeniid cestode zoonoses: cystic echinococcosis, alveolar echinococcosis and neurocysticercosis. Adv Parasitol. 1996;38:169–250. 10.1016/S0065-308X(08)60035-4 8701796

[7] Macpherson CN, Kachani M, Lyagoubi M, Berrada M, Shepherd M, Fields PF, Cystic echinococcosis in the Berber of the Mid Atlas mountains, Morocco: new insights into the natural history of the disease in humans. Ann Trop Med Parasitol. 2004;98:481–90. 15257798 10.1179/000349804225021343

[8] Macpherson CN, Bartholomot B, Frider B. Application of ultrasound in diagnosis, treatment, epidemiology, public health and control of *Echinococcus granulosus* and *E. multilocularis.* Parasitology. 2003;127(Suppl):S21–35. 10.1017/S0031182003003676 15027603

